# An all-Optical Photoacoustic Sensor for the Detection of Trace Gas

**DOI:** 10.3390/s20143967

**Published:** 2020-07-16

**Authors:** Thomas Lauwers, Alain Glière, Skandar Basrour

**Affiliations:** 1 Université Grenoble Alpes, CEA, LETI, F38000 Grenoble, France; alain.gliere@cea.fr; 2 Université Grenoble Alpes, CNRS, Grenoble INP, TIMA, 38000 Grenoble, France; skandar.basrour@univ-grenoble-alpes.fr

**Keywords:** Extrinsic Fabry-Perot interferometer, photoacoustic sensor, trace gas sensor, lumped model, all-optical sensor, all-optical PA sensor, multipass PA sensor

## Abstract

A highly sensitive Fabry–Perot based transduction method is proposed as an all-optical alternative for the detection of trace gas by the photoacoustic spectroscopy technique. A lumped element model is firstly devised to help design the whole system and is successfully compared to finite element method simulations. The fabricated Fabry–Perot microphone consists in a hinged cantilever based diaphragm, processed by laser cutting, and directly assembled at the tip of an optical fiber. We find a high acoustic sensitivity of 630 mV/Pa and a state-of-the-art noise equivalent pressure, as low as $\sim \;2\;\mu \mathrm{Pa}/\sqrt{\mathrm{Hz}}$ at resonance. For photoacoustic trace gas detection, the Fabry–Perot microphone is further embedded in a cylindrical multipass cell and shows an ultimate detection limit of 15 ppb of NO in nitrogen. The proposed optical trace gas sensor offers the advantages of high sensitivity and easy assembling, as well as the possibility of remote detection.

## 1. Introduction

The photoacoustic (PA) effect consists in the generation of an acoustic wave originating from the specific absorption of laser radiation by the medium of interest. Based on this phenomenon, the photoacoustic spectroscopy technique enables high sensitivity probing [1,2] and sensing [3,4]. The targeted molecules absorb a part of the optical energy emitted from the laser and release it by thermal relaxation. The modulation of the optical power or wavelength creates a variation of the heat produced, acting as a harmonic source for the photoacoustic pressure wave, whose amplitude indicates the targeted molecules concentration. For gas sensing, the mid-infrared range, often called the molecule fingerprint region, is a very interesting zone for photoacoustic spectroscopy, where most molecules of interest (polluting or toxic gases, biomarkers, etc.) have a specific spectral signature.

A generic trace gas photoacoustic sensor can be composed of a laser, a closed volume, called the cell, where the acoustic wave is generated, and a transducer for the detection of the pressure variation. Most systems use electromechanical transductions. Among them, the standard condenser microphone or the piezoelectric quartz tuning fork are known for their robustness, high stability, low-cost and miniaturization capabilities. Due to the low pressure level generated by PA effect, the signal is often amplified by means of acoustic [5], mechanical, [6,7], or optical [8,9] resonance. 

Optical readout of the acoustic wave can be an interesting alternative to electromechanical transductions for PA detection in harsh environment, where high temperatures or electromagnetic noise can severely damage the transducer or decrease its sensitivity. A wide variety of optical transductions have already been proposed in the literature, ranging from the simple position sensor [10] to interferometric readout systems, like Mach Zehnder [11] or free space or fibered Michelson configurations [12,13]. However, those systems are bulky and require either a precise alignment of the optical elements or long optical fibers, causing a loss of robustness. Among the interferometric transducers, a variant of the traditional Fabry–Perot (FP) cavity, called fiber-optic extrinsic Fabry–Perot interferometer (EFPI) [14,15], can settle the size problem by using the air/fiber interface as first mirror and a reflective diaphragm as second mirror. This low finesse optical cavity, requiring no specific alignment, enables remote detection with optical fiber [16]. Different materials and geometries have been proposed, involving mainly silver [17], graphene [18] or parylene [19] circular diaphragms, or more recently stainless steel cantilevers [20]. The latter are more sensitive, due to the enhanced displacement obtained at the free end. The photoacoustic signal in such systems is mainly amplified by means of mechanical resonances, sometimes coupled with optical amplification, as has been shown in the recent work by Zhang et al. [21], with the use of Heriott cells. 

In this paper, we develop a lumped element model to assist in the design of an EFPI based photoacoustic sensor. Compared to the finite element methods (FEM), this analytical model provides much faster problem solving with good predictive capability. The second part of the article is focused on the experimental implementation of such a sensor. We propose a highly sensitive all-optical PA sensor based on an EFPI transducer. The latter builds on a previous work on the resonant hinged cantilever [22], with enhanced sensitivity through an improved laser cutting process and the use of a thicker structure. To create the PA sensor, the transducer is embedded in a cylindrical multipass cell, thus increasing the interaction length between the light and the gas of interest. We finally present a complete characterization of our all-optical PA sensor prototype and assess the sensor performance on a weak NO absorption line.

## 2. Design of the Photoacoustic Sensor

### 2.1. Description of the System

**Figure 1:**
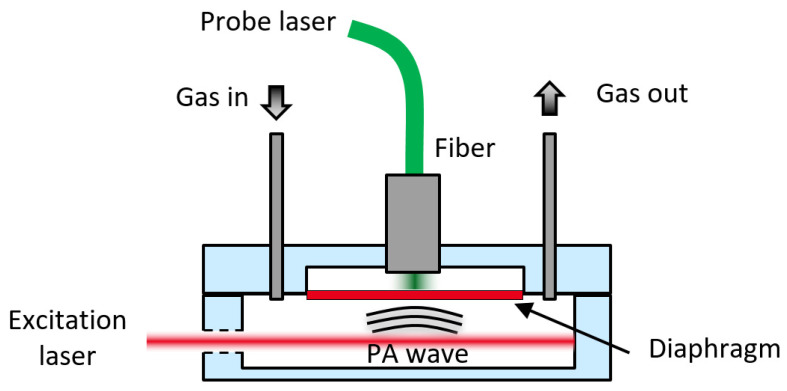
Diagram (not to scale) of the all-optical multipass PA trace gas sensor with the PA cell (lower volume), the transducer part with the optical cavity (upper volume), the probe and excitation laser (respectively green and red light beam).

The all-optical photoacoustic sensor proposed here (Figure 1) is composed of a cylindrical cell filled with gas, illuminated (red light beam) by a mid-infrared quantum cascade laser (QCL) to generate the acoustic wave (lower, front volume). The pressure wave generated is measured by the fiber optic Fabry–Perot acoustic transducer (upper, back volume), with a second laser diode at 1.55 µm (green light beam) used to probe the position of a reflective diaphragm sensitive to acoustics. 

**Figure 2:**
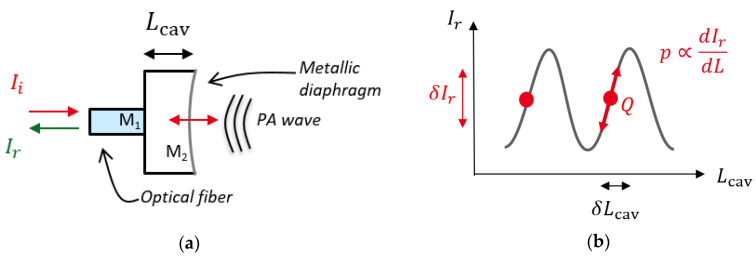
(**a**) Diagram of the fiber-optic Fabry–Perot based transducer; (**b**) interferometric pattern of the reflected optical signal with quadrature point Q.

The transducer (Figure 2a) is composed of two mirrors. The first one (M_1_), with low reflectivity, is constituted by the air/fiber interface and the second one (M_2_) is a reflective mirror, designed to be highly sensitive to acoustic perturbations. The light emitted by a 1.55 µm laser diode is injected in the FP cavity through an optical fiber and the reflected light is measured by a photodetector. 

The reflectivity of the FP cavity $R_{\mathrm{FP}}$ can be analytically calculated considering a two light—beam interferometer:

(1) $$R_{\mathrm{FP}}\left(\lambda ,L_{\mathrm{cav}}\right)=R_{1}+R_{2}+2\sqrt{R_{1}R_{2}}\cos\left(\frac{4\pi L_{\mathrm{cav}}n}{\lambda }\right).$$

**Figure 3:**
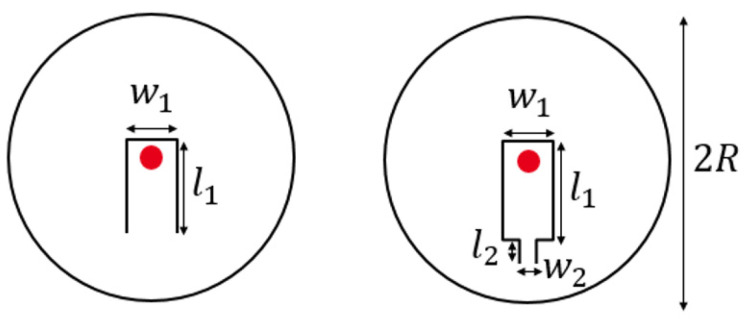
Diagram of the cantilever-based diaphragm (**left**) and the hinged cantilever-based diaphragm (**right**). Red spots indicate the probe laser location.

Here $R_{1}$ and $R_{2}$ correspond respectively to the reflectivity of the air/fiber interface and the reflective diaphragm, $L_{cav}$ is the length of the FP cavity, n is the refractive index of the medium and λ is the wavelength of the probe laser. With the assumption that the laser wavelength λ is stable and that the refractive index variations are slow with respect to the acoustic frequency, the optical power reflected back from the Fabry–Perot cavity oscillates according to the variation $\delta L_{cav}\left(p\right)$ of the length of the optical cavity:

(2) $$\delta I_{r}\left(p\right)=I_{i}\frac{dR_{\mathrm{FP}}}{dL}\delta L_{cav}\left(p\right)\sin\left(\omega_{ac}t\right),$$

where $I_{i}$ is the incident power of the laser, p the amplitude of the photoacoustic pressure wave and $\omega_{ac}$ the acoustic angular frequency. To obtain a linear relation between the displacement and the reflected power with high sensitivity, the probe laser wavelength is set to the quadrature point (point Q on Figure 2b). The displacement $\delta L_{cav}$ induced by the pressure wave depends on the mechanical structure and the photoacoustic cell geometry. Due to the cylindrical symmetry of the fiber, the diaphragm is a circular plate clamped on its circumference. To beneficiate from a large displacement of the structure, it has been decided to embed a cantilever structure in the plate (Figure 3), so that the free end of the cantilever is situated at the center, where the probe laser thus measures the maximum deflection (red spot on Figure 3).

The problem arising for this particular structure is the coupling between the unwanted damped plate resonance and the sensitive cantilever resonance. In order to decouple these mechanical modes we propose a hinge geometry (Figure 3, right) detailed in the next section. Moreover, this structure allows us to obtain a resonant frequency in the kHz range while using a relatively thick structure (100 µm) which is easy to fabricate and assemble.

### 2.2. Mechanical Model

The mechanical structure is designed to resonate at a few kHz, well below the cut-off frequency induced by the relaxation time of the molecules of interest (few tens of kHz for simple molecules, such as NO, in air [23]). In order to simplify the study of the diaphragm’s dynamics, it is possible to isolate the clamped-free cantilever and the clamped circular plate fundamental resonance modes, respectively calculated with the Euler beam and Kirchhoff—Love plate theories:

(3) $$\omega_{m,c}=\alpha_{c}\frac{t_{m}}{l_{1}^{2}}\sqrt{\frac{E}{12\rho }}\;\;\;\;\;\;\;\;\;\;\mathrm{and}\;\;\;\;\;\;\;\;\omega_{m,p}=\alpha_{p}\frac{t_{m}}{R^{2}}\sqrt{\frac{E}{12\rho \left(1-\nu^{2}\right)}}.$$

Here $\omega_{m,c}$ (respectively $\omega_{m,p}$) refers to the first mechanical resonance angular frequency of a clamped-free cantilever (respectively a clamped circular plate), $\alpha_{c}=3.52$ and $\alpha_{p}=10.22$ are the coefficients corresponding to the fundamental modes of resonance, $t_{m}$ is the thickness of the structure, $l_{1}$ the length of the cantilever, R the radius of the plate, $E_{m}$ the Young modulus of the material, $\rho_{m}$ its mass density and $\nu_{m}$ its Poisson’s coefficient.

The hinged cantilever resonant angular-frequency $\omega_{m,\mathrm{hc}}$ can also be calculated, by the Rayleigh’s quotient method [24]:

(4) $$\omega_{m,\mathrm{hc}}=\sqrt{\frac{w_{2}\left[l_{2}\left(3l_{1}^{2}+3l_{1}l_{2}+l_{2}^{2}\right)w_{1}+l_{1}^{3}w_{2}\right]}{B}}t_{m}\sqrt{\frac{E_{m}}{\rho_{m}}},$$

where $w_{1}$, $l_{1}$ refer to the width and length of the cantilever, $w_{2}$, $l_{2}$ to the width and length of the hinge, and B is a polynomial function of the cantilever geometry (for more details, see Appendix A).

One way to ensure that the first bending mode of the cantilever of the coupled structure will not be disturbed by the fundamental plate mode is to ensure that $\omega_{m,p}\gg \omega_{m,c}$. In this way, only the higher cantilever mode orders will be coupled with the plate. With this condition fulfilled, we also expect a correct prediction of the isolated case on the final system dynamics. To identify a suitable cantilever length $l_{1}$ we calculate the resonance frequency of the three structures and we introduce the reduced frequency x defined as the ratio $x=\omega_{m,c}/\omega_{m,p}$. 

**Figure 4:**
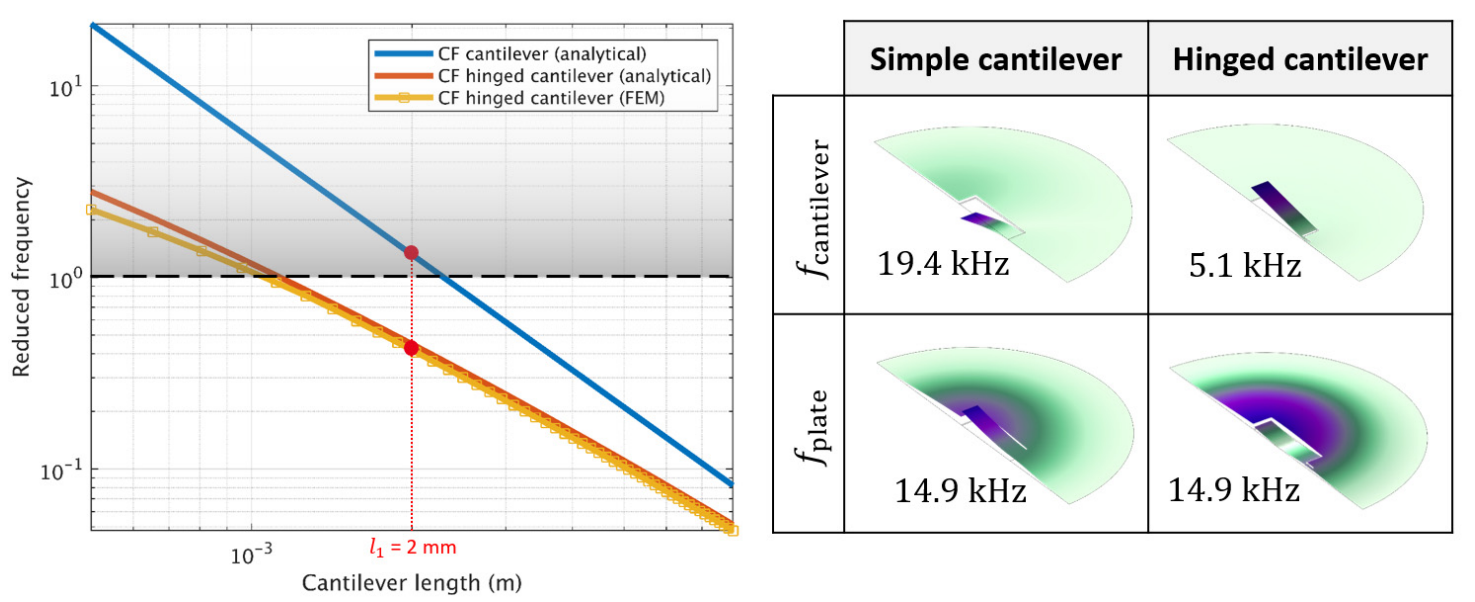
On the left, reduced frequency of the isolated cantilever beam (blue) and hinged cantilever beam (analytical model in red and FEM result with yellow squares) as a function of the length $l_{1}$. On the right, first two eigenmodes of the cantilever-based diaphragm (first column) hinged cantilever-based diaphragm (second column).

For the calculations, a plate thickness $t_{m}=100\;\mu m$ with a radius R=4 mm are fixed to obtain a high plate resonance frequency and to meet with the integration constraints of the diaphragm inside the photoacoustic cell. Then we chose a cantilever width $w_{1}=1\;\mathrm{mm}$ and the hinge parameters $w_{2}=100\;µm$ and $l_{2}=300\;µm$. This set of parameters allows to obtain a flexible hinge and gives a large flexibility for the choice of $l_{1}$ while remaining feasible by the diaphragm fabrication process. The material parameters are $E_{m}=185\;\mathrm{GPa}$ and $\rho_{m}=7850\;\mathrm{kg}/m^{3}$. Figure 4 compares the reduced frequency for the case of the normal clamped-free (CF) cantilever (blue curve) and the hinged CF cantilever (red curve). The hinged cantilever resonance frequency calculated with the Rayleigh’s quotient method is very close to the finite element method (FEM) results (yellow curve) performed with a commercial software (COMSOL Multiphysics, COMSOL AB, Sweden). We clearly see on the figure that adding a hinge helps to lower the cantilever resonance frequency and thus to lie in the zone where x<1. For a length of $l_{1}=2\;\mathrm{mm}$, the analytical calculation gives $x_{\mathrm{cantilever}}\;\sim \;1$ while $x_{\mathrm{hinged}\text{-}\mathrm{cantilever}}\;\sim \;0.3<1$, enabling a weak coupling between the hinged cantilever and the plate mode. 

This result is confirmed by taking in account the complete mechanical structure with a FEM eigenmode study, presented on the right side of Figure 4. As expected, the eigenmode resolution for the simple cantilever based diaphragm (first column) shows a first cantilever mode (19.4 kHz) that occurs after the fundamental plate mode (x>1), while for the hinged cantilever based diaphragm (second column) the first cantilever mode (5.1 kHz) is well below the fundamental plate mode (x ~ 0.3).

However, the cantilever mode of the complete structure differs notably from the isolated cantilever mode: this is because the cantilever is not perfectly clamped at the hinge end, which is situated at around 2.5 mm from the clamped circumference. The analytical calculation of the isolated hinged cantilever resonance frequency gives $f_{m,\mathrm{hc}\;}=6.7\;\mathrm{kHz}$ which is above the frequency obtained for the complete structure. To match the isolated case resonance frequency and the one obtained with the FEM resolution on the complete structure, an effective hinge length $l_{2,\mathrm{eff}}$ is introduced in the mechanical resonance formula from Equation (4), which is found to be 480 μm instead of 300 μm.

### 2.3. Photoacoustic Model

To provide a complete description of the PA sensor, the next section focuses on the photoacoustic generation and the acoustic coupling between the considered medium (ambient air or nitrogen in our case) and the mechanical structure. In the following sections, the fluid is considered as an ideal gas with equilibrium pressure, temperature and velocity $P_{0},\;T_{0},\;v_{0}$ and described by small variations of the pressure field p, temperature τ and velocity v, which are a solution of the fully linearized Navier–Stokes equations (FLNS).

#### 2.3.1. Heat Source

The generation of the photoacoustic wave originates from a periodic heat source due to the specific absorption of laser radiation. Using the energy equation of the FLNS model, the temperature fluctuation inside the PA cell at the position r=(r,θ,z) and angular frequency ω can be expressed as:

(5) $$\tau \left(r,\omega \right)=\left(1-F\left(r,\omega \right)\right)\frac{1}{\rho_{0}c_{P}}\left[p+\frac{Q}{𝕛\omega }\right],$$

where Q refers to the deposited power density in $W.m^{-3}$, $\rho_{0}$ the air density and $c_{p}$ the heat capacity at constant pressure. F is a function taking into account the thermal loss originating from conduction [25] and is a solution of the Helmholtz equation. The temperature expression obtained in Equation (5) can be introduced in the FLNS continuity equation in the integral form:

(6) $$\int_{V}\left(𝕛\omega \frac{p}{P_{0}}\right)dV=\int_{\partial V}v.\left(-n\right)dS+\int_{V}\left(𝕛\omega \frac{\tau }{T_{0}}\right)dV,$$

where V indicates the volume of the cell and n is the outward pointing unit vector, normal to the volume boundary ∂V. If we consider a closed cell with volume $V_{fv}$ that is small enough to have a uniform pressure distribution, the equation (6) leads after integration to:

(7) $$p=i_{PA}Z_{fv}\left(\omega \right)\;\mathrm{with}\;Z_{fv}\left(\omega \right)=\frac{1}{𝕛\omega C_{fv}}\;\frac{1-\langle F\rangle }{\langle F\rangle \gamma +\left(1-\langle F\rangle \right)}\;\mathrm{and}\;i_{PA}=\frac{\left(\gamma -1\right)}{C_{fv}}Q,$$

where γ is the heat capacity ratio, $C_{fv}=V_{fv}/\left(\gamma P_{0}\right)$ is called acoustic compliance of the cell, ⟨F⟩ depends on ω and refers to the volume averaged value of F(r,ω). This equation can be represented by an electric equivalent model, where the heat source Q is responsible for gas expansion and acts as a current source $i_{PA}$ ($m^{3}.s^{-1}$), while the pressure increase p depends on the impedance $Z_{fv}\left(\omega \right)$. The analytical expression of $Z_{fv}\left(\omega \right)$ is not straightforward because it is expressed with the ⟨F⟩ function which cannot be simply solved in the case of a cylindrical volume. The approach used here consists in considering two asymptotic cases of the cylindrical cell of radius $R_{cell}$ and height $h_{cell}$. The thermal loss occurring on the cylinder sidewalls (denoted $R_{r}$) is obtained by considering an infinitely long cylinder ($R_{cell}\ll h_{cell}$) and by evaluating the real part of $\underset{\omega \to 0}{\lim\;}Z_{fv}\left(\omega \right)$. In a similar way, the loss occurring on the cylinder ends (denoted $R_{z}$) is obtained by considering an infinitely flat cylinder ($R_{cell}\gg h_{cell}$). The analytical expressions of $R_{r}\;\mathrm{and}\;\;R_{z}$ are given in Appendix B. Combining the two asymptotic cases gives a final thermal resistance $R_{th}$, corresponding to $R_{r}$ in parallel with $R_{z}$. The cell impedance can then be written in term of the thermal resistance in parallel with the acoustic compliance $C_{fv}$:

(8) $$R_{th}=\frac{\rho_{0}c_{p}P_{0}}{8\kappa \pi }\frac{h_{cell}}{h_{cell}^{2}+3/2R_{cell}{}^{2}}\;\mathrm{and}\;C_{fv}=\frac{V_{fv}}{\gamma P_{0}}.$$

**Figure 5:**
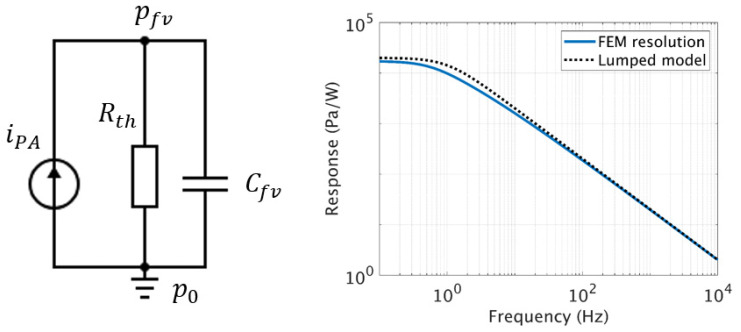
On the left, equivalent electrical circuit responsible for the pressure generation in the closed front volume p_fv_. On the right, transfer function of the circuit (generated pressure per deposited power) and comparison with the FEM simulation using the thermoacoustic model.

The photoacoustic pressure generation thus behaves like a low-pass filter, with a cut-off angular frequency $\omega_{h}=1/R_{th}C_{fv}$. This effect is due to the coupled effect of the volume and the thermal resistance taking into account the losses due to thermal conduction to the walls. The Figure 5 shows the equivalent circuit with the current source and a comparison between the lumped model and a FEM resolution of the FLNS equation in the case of a cylinder with a radius $R_{cell}=10\;\mathrm{mm}$ and a height $h_{cell}=10\;\mathrm{mm}$. These dimensions correspond to the size of the already available PA cell used for the experimental implementation.

With the lumped model we can calculate the transfer function corresponding to the increase of pressure for a given deposited power in Pa/W. We observe a capacitive behavior of the pressure inside the cell volume $p_{fv}$ at the working frequencies from 1 kHz to 10 kHz, while a plateau is noticeable at very low frequencies below 1 Hz, due to the thermal losses $R_{th}$. Both asymptotic behaviors are correctly described by the analytical lumped model.

#### 2.3.2. Acoustic Coupling

**Figure 6:**
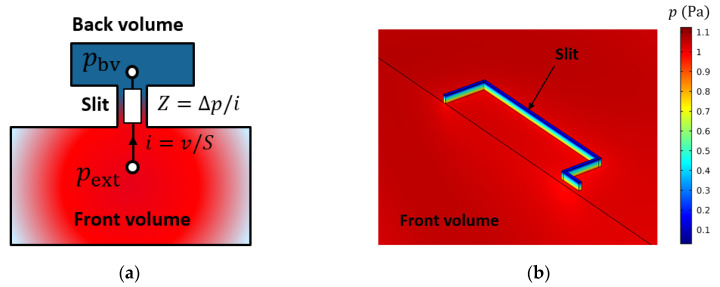
(**a**) Sectional schematic of the slit problem; (**b**) Pressure behavior inside the slit and the front volume for a slit length of 100 µm with $p_{ext}=1\;\mathrm{Pa}$. (the mechanical structure and the back volume are not shown for the sake of clarity).

Once the photoacoustic transfer function is defined the behavior of the fluid surrounding the diaphragm must be described. The problem is divided in three parts: the external environment, the ventilation slit, which corresponds to the gap between the cantilever frame and the rest of the plate, and the back volume constituting the FP cavity (Figure 6a). The diaphragm is supposed to be rigid (no displacement). By reducing the three FLNS equations to a one-dimensional slit problem we can find an analytical solution of the pressure propagation as a solution of the Helmholtz equation [26]:

(9) $$\frac{\partial^{2}p}{\partial z^{2}}+k_{l}{}^{2}p=0$$

where $k_{l}$ is a thermoviscous wave vector that depends on the air parameters and slit geometry. This harmonic behavior can be formulated with a lumped element model, where the differential pressure across the slit is described with a thermoviscous impedance $Z_{v}$. In a similar way to the previous section, $Z_{v}$ is defined as the ratio between the differential pressure between both parts of the slit $\Delta p=p_{bv}-p_{ext}$ and the fluid velocity flow through the slit, normal to the diaphragm surface, denoted i=v S (see Figure 6a), where S is the ventilation slit area. In the slit configuration, $Z_{v}$ is mainly dominated by its real part, which represents the viscous resistance $R_{v}$, that takes in account the viscous losses experienced by the fluid through the thin slit. On the other hand, the volume of the back chamber introduces an acoustic compliance $C_{bv}$ (see Section 2.3.1):

(10) $$R_{v}=12\mu_{0}\frac{t}{w_{v}^{3}l_{c}}\;\mathrm{and}\;C_{bv}=\frac{V_{bv}}{\gamma P_{0}}.$$

Here $\mu_{0}\;\mathrm{and}\;P_{0}$ correspond to the viscosity and atmospheric pressure, and $V_{bv}$ is the volume of de cavity. The cantilever slit is assimilated to a rectangular slit with a width $w_{v}$ and a length $l_{c}$ equal to the perimeter of the hinged cantilever: $l_{c}=2(l_{1}+l_{2})+2w_{1}-w_{2}$. 

A FEM resolution of the FLNS equation is performed to confirm the results obtained with the lumped model for the acoustic response of the transducer. Here, the external environment consists in a harmonic plane wave boundary condition, the mechanical structure is assimilated to a rigid wall and the back volume has isothermal walls boundary conditions. The slit has the shape of the hinged cantilever, a height of 100 µm and a width of 35 µm. The volume of the slit surrounding the hinged cantilever is reported on Figure 6b. We can note the pressure variation along the height of the slit from 0.1 Pa in the back volume up to 1.1 Pa in the front volume. 

Coupled to the back volume, this impedance induces a high-pass filter, meaning that the fluid leaks through the slit at low frequencies and thus slowly equilibrates the differential pressure between both sides of the diaphragm. 

**Figure 7:**
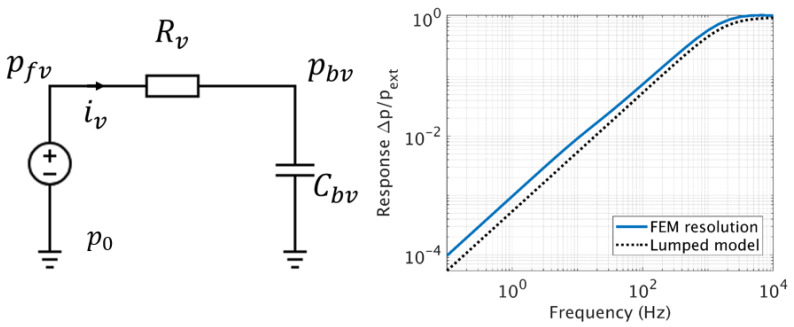
On the left, equivalent electrical circuit responsible for the pressure differential across the slit, given an applied pressure p_ext_. On the right, transfer function of the circuit and comparison with the FEM simulation using the FLNS model.

As can be seen from the Figure 7, a good agreement is obtained between the FEM simulations and the lumped element model. The acoustic response of the system shows that up to a certain cut-off frequency $f_{v}$, the gas leak through the slit diminishes, leading to an increase of the differential pressure and thus the measurable signal. Therefore, the slit should be chosen small enough to give a cut-off frequency $f_{v}$ situated below the mechanical resonance frequency of the system, denoted $f_{m}$. 

### 2.4. Global Resolution of the System

**Figure 8:**
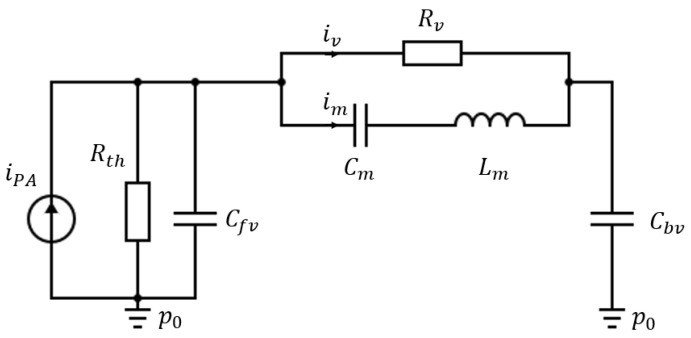
Complete equivalent electrical circuit of the PA sensor with (from left to right) the current source and the front volume, the acoustic slit with a velocity flow $i_{v}$ and the mechanical diaphragm with a velocity flow $i_{m}$ and, finally, the back volume.

The complete lumped elements model, represented on Figure 8, can be used to describe quantitatively the harmonic response of the photoacoustic sensor. 

The mechanical parameters $C_{m},L_{m}$ have been calculated with the expression of the stiffness and mass (see Appendix A) and the effective hinge length calculated in Section 2.2:

(11) $$\{\begin{array}{c} C_{m}=S_{\mathrm{eff},m}^{2}/k_{\mathrm{eff},m}\\ L_{m}=m_{\mathrm{eff},m}/S_{\mathrm{eff},m}^{2} \end{array},$$

where $S_{\mathrm{eff},m}$ is the effective area of the cantilever given in Appendix A. The thermoacoustic parameters $R_{th},\;C_{fv},\;\;R_{v},\;\;C_{bv}$ have been calculated with the formula from Section 2.3. The fluid flow induced by the heat source $i_{PA}$ is calculated for a deposited power of 1 mW, and the cantilever displacement Δz is given by the potential difference $\Delta p_{C}$ across the mechanical compliance $C_{m}$, as $\Delta p_{C}=i_{m}/\;\left(𝕛C_{m}\omega \right)=\left(\Delta zS_{\mathrm{eff},m}\right)/C_{m}$.

**Figure 9:**
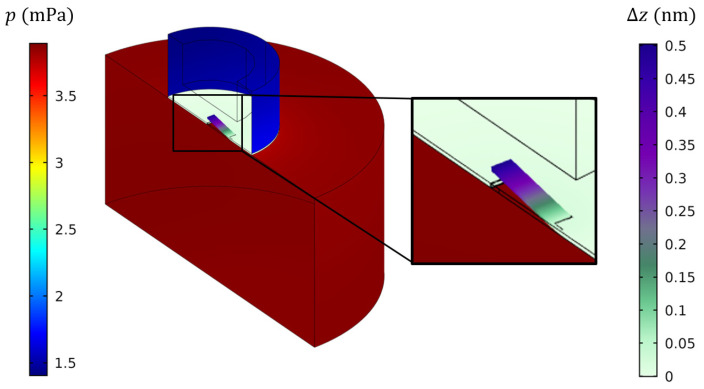
Results of the FEM simulations for the complete PA sensor near the resonance frequency for a deposited power of 1 mW. The left color bar indicates the pressure value in the front volume (dark red) and back volume (blue), while the right color bar indicates the displacement of the cantilever.

On the other hand, a FEM simulation taking into account the precise geometry of the system can be implemented. The front and back volumes are surrounded with isothermal walls and are separated from each other by the mechanical structure, modelled by a 3D plate. Finally, the laser excitation is modelled by a uniform source term distributed in the entire front volume. The two color maps on Figure 9 indicate the pressure field amplitude in the front and back volume, and the displacement amplitude of the cantilever. Let us note that the small volume approximation made in the previous section to establish the lumped element model is verified here, since the pressure fields in both volumes are uniform.

**Figure 10:**
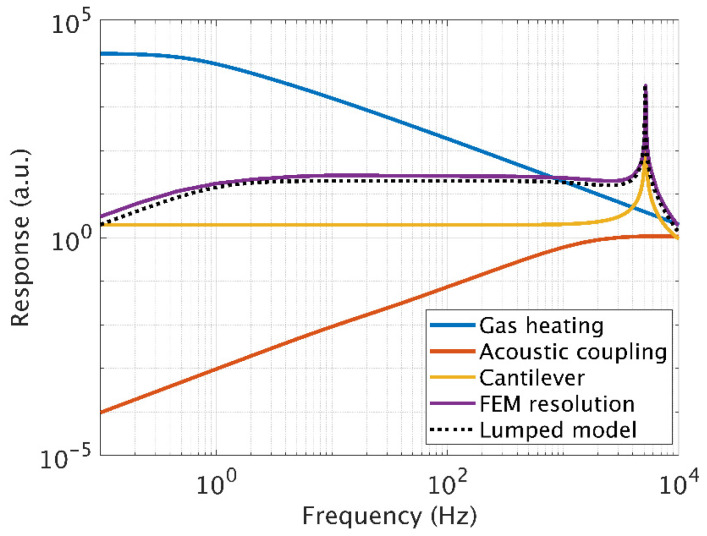
Calculated transfer function of the different blocks of the PA sensor and complete transfer in nm/W with FEM and comparison with the lumped element model (dotted black curve).

Figure 10 shows the transfer function obtained by FEM resolution of the FLNS model for the different blocks studied: the blue curve corresponds to the photoacoustic generation (Section 2.3.1), the red curve to the acoustic transfer of the transducer (Section 2.3.2) and the yellow curve shows the mechanical transfer function of the diaphragm (Section 2.2). Finally, the FEM resolution taking in account the fluidic and mechanical coupling in purple is expressed in nm/W (cantilever displacement per deposited unit of power) and compared to the transfer obtained by analytical resolution of the lumped elements model (dotted black). A very good agreement between the analytical model and the FEM simulation is obtained.

Based on the modelling and simulation results, a set of rules can be established for further designs. Firstly, it is advantageous to consider a small ventilation slit width to obtain the condition $\omega_{v}<\omega_{m}$. Secondly, from Section 2.3.1, it appears that a reduced height $h_{cell}$ leads to a decrease of the energy lost by conduction mechanism through the walls ($R_{th}$), meaning a higher value of $\omega_{h}$, and thus an increase of the generated photoacoustic pressure at the working frequency $f_{m}$. Finally, a large cylinder radius is preferred, in order to increase the gas-laser light interaction and consequently the deposited power in the PA cell.

## 3. Fabrication and Experimental Set-Up

### 3.1. Acoustic Transducer

**Figure 11:**
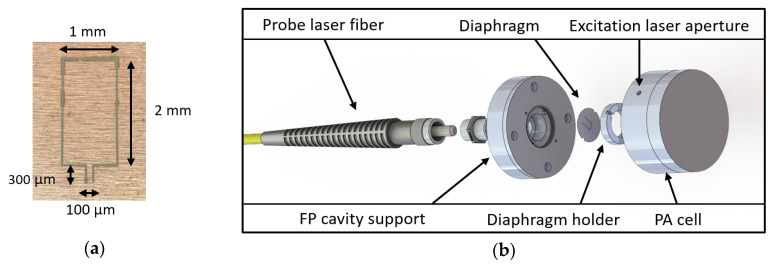
(**a**) Microscope image of the manufactured hinged cantilever; (**b**) 3D view of the fabricated PA sensor with (from left to right) the optical fiber, a threaded adapter, the FP transducer support, the cantilever-based diaphragm, a threaded ring to hold the diaphragm and the cylindrical multipass PA cell.

The diaphragm is fabricated from a 100 µm thick stainless-steel foil with laser micromachining process. A water jet laser technology (Synova SA, Duillier, Switzerland) has been employed to obtain flat structures with reduced residual stress (Figure 11a). The minimal cutting size is around 35 µm. This sets the ventilation slit width around the cantilever and induces a cut-off frequency around $f_{v}=1.3\;\mathrm{kHz}$. 

The acoustic transducer part is made with an aluminum support, which is simply screwed onto a fiber (Figure 11b). To enable fast prototyping and test of different mechanical resonator geometries the diaphragm is aligned by two posts and held on the support with a screwed ring to obtain a circularly clamped condition.

The last element of the sensor is the photoacoustic cell, which is fixed to the acoustic transducer. A cylindrical metallic cell available “off-the-shelf” was used, with a 10 mm radius and a height of 10 mm. Its walls have been polished in order to achieve maximum reflections and thus maximize the length of the laser interaction with the gas. Finally, a tapered aperture is drilled in the cylinder to let the excitation laser light enter without exit. 

### 3.2. Experimental Bench Set-Up

**Figure 12:**
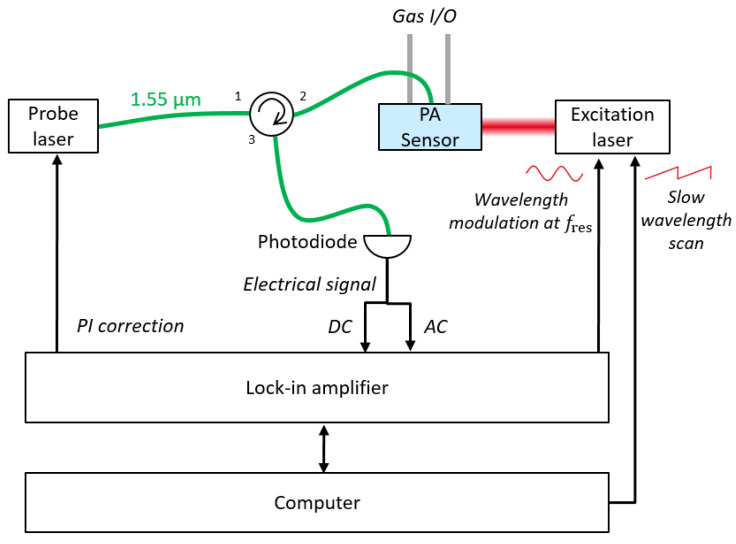
Diagram of the photoacoustic measurement setup. Optical fibers are drawn in green and electric connections in black.

The all-optical PA sensor has been used to detect traces of NO in N_2_, with the following experimental set-up (Figure 12). The probe DFB laser (Eblana photonics, Ireland), emitting at 1.55 µm, is launched into the fiber optic FP cavity. The reflected light is retrieved with an optical circulator and measured by a photodiode with a high frequency response (500 kHz). The pump laser is a Quantum Cascade Laser (QD5250CM1, Thorlabs Inc, Newton, NJ, USA) with a power of 100 mW emitting in the mid-infrared at 5.2 µm, where a weak NO absorption peak lies (absorbance of $1.7\times 10^{-5}\;{\mathrm{cm}}^{-1}$ for 1 ppm of NO). The QCL is stabilized in temperature and the current is controlled for a slow wavelength scan across the NO absorption peak and a wavelength modulation scheme [27]. The NO is injected in the PA sensor through 1/4″ tubes from a calibrated gas cylinder containing 25 ppm of NO in N_2_. The injection and dilution are performed with a gas diluter (GasMix, Alytech, Juvisy-sur-Orge, France).

The wavelength modulation is performed with a lock-in amplifier (HF2LI, Zurich Instrument, Switzerland). The AC part of the signal retrieved from the photodetector is demodulated at the same frequency for a 1f detection or at twice the modulation frequency for a 2f detection. While the 1f signal has the greatest amplitude and is used to determine the location of an absorption peak, the 2f detection method is preferred for gas calibration as it allows eliminating the background signal. 

Due to the high sensitivity of the FP cavity, the location of the quadrature working point can be perturbed by environmental parameters like temperature, static pressure or humidity. This leads to a drift of the working point and as a consequence a time variation of the sensitivity. In order to stabilize this drift, a proportional integral (PI) correction is implemented to control the emission wavelength of the probe laser and to keep the DC optical signal at its quadrature value. The tuning of the wavelength is directly obtained by controlling the drive current of the laser. This eliminates the need for an expensive and bulky piezo controlled external cavity diode laser, but results in unwanted variation of the emitted power, which is also a function of the drive current. In order to remain insensitive to the emitted power variations, the DC signal, on which the PI correction is made, is normalized with respect to the power of the probe laser. If the drift reaches the upper (or lower) limit of the laser wavelength tunability, a new quadrature point, lying in the laser tuning range, is recycled [28]. This closed loop operation helps to obtain a long-term stability of the system, as well as high sensitivity and immunity to low frequency external perturbations.

## 4. Experimental Results

The complete characterization of the sensor has been performed in two steps. Firstly, the acoustic sensitivity of the FP transducer alone has been measured by using it as a microphone, placed in front of a calibrated speaker. Secondly, the FP transducer has been mounted on a multipass photoacoustic cell and, with the help of a commercial gas diluter, further characterized (resonance frequency, NO limit of detection and Allan deviation).

### 4.1. Microphone Characterization

**Figure 13:**
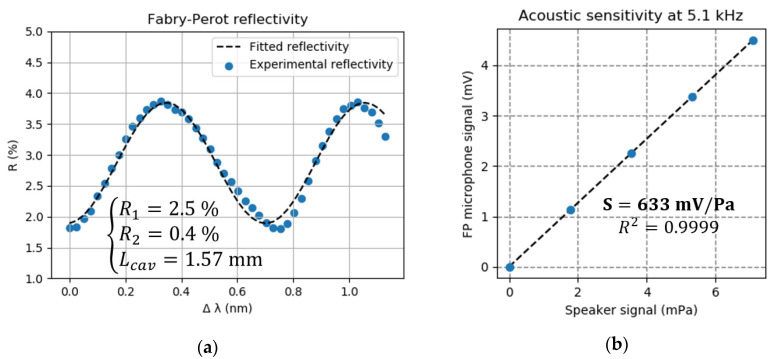
(**a**) Interference pattern of the transducer FP cavity as a function of the relative wavelength shift Δλ around 1.55 µm; (**b**) Acoustic calibration of the FP transducer at the hinged cantilever resonance frequency (5.1 kHz).

During this first stage of characterization, the acoustic transducer is placed in an insulation box, together with a calibrated speaker, to assess the microphone operation mode. Before each measurement, an initialization step is carried out as follows to determine the optimal working point. A sweep on the drive current of the probe laser is made to scan a few periods of the Fabry-Perot interferences. After a normalization by the emitted power, the signal is fitted to a sinusoid in order to retrieve the phase shift, the interfringe period and fringe amplitude and offset. The quadrature working point can then be calculated and is used to lock the DC signal, using a PI correction loop. Figure 13a shows the retrieved reflectivity of the cavity and the sinusoidal fit using the analytical FP reflectivity of Equation (1). In abscissa, the wavelength shift is relative to the working wavelength of 1.55 μm. The reflectivity of the first mirror $R_{1}$ corresponds to the air/fiber interface, while the second reflectivity $R_{2}$ takes into account not only the reflectivity of the cantilever but also the overall losses of this low-finesse cavity (divergence of the fiber output, misalignment of the diaphragm, etc.).

Figure 13b shows the acoustic sensitivity measured at the resonance frequency of the hinged cantilever (5.1 kHz). Each point is calculated as the mean value for a time duration of 10 s and is associated with its standard deviation (too small to be represented on the figure). We observe a linear acoustic response and a high sensitivity of 630 mV/Pa. Given the standard deviation obtained when no acoustic signal is applied and a measurement bandwidth of 1 Hz, a LOD of $\sim \;2\;\mu \mathrm{Pa}/\sqrt{\mathrm{Hz}}$ is obtained. The LOD is probably overestimated as it corresponds in fact to the white noise present in the experiment room, which is not completely attenuated by the homemade acoustic insulation box used.

### 4.2. Photoacoustic Measurements 

**Figure 14:**
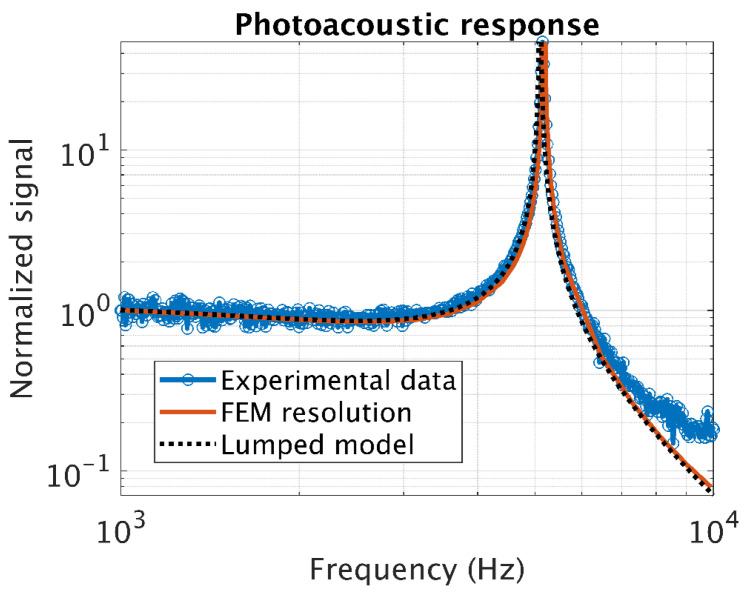
Normalized frequency response of the PA sensor in presence of atmospheric water at 1906 cm^−1^.

The FP transducer has then been mounted on a multipass cell for further characterization in photoacoustic detection mode. To identify the resonance frequency $f_{\mathrm{res}}$ in the closed PA cell, the response of the transducer is probed on a strong atmospheric water absorption line at 1906 cm^−1^. To do this, the wavelength of the QCL is modulated around the absorption peak. The wavelength modulation is realized via the drive current of the QCL, with an amplitude of 10 mA corresponding to a modulation depth 0.16 cm^−1^, and a frequency varying from 1 to 10 kHz. The normalized response (Figure 14) shows an excellent agreement with the FEM model with a resonant frequency of 5.16 kHz and a quality factor of 70 in the closed photoacoustic cell. 

**Figure 15:**
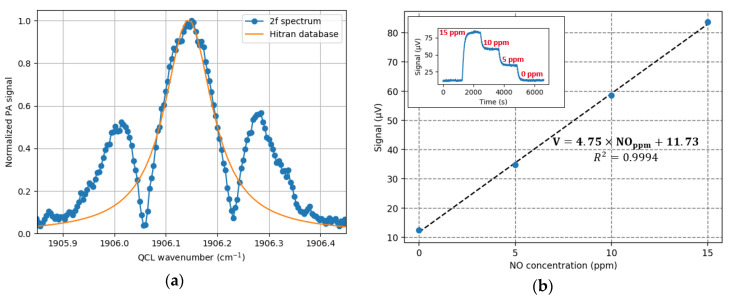
(**a**) 2f scan of the NO absorption line (blue) compared with the Hitran database (orange); (**b**) Calibration of the PA sensor for a measurement of 6600 s.

Once the exact resonance frequency is known, a second harmonic detection (2f) strategy is used to identify the NO absorption peak [27]. In the 2f wavelength scheme, the modulation frequency is at $f_{\mathrm{res}}/2$ and the signal demodulated at $f_{\mathrm{res}}$. As shown on the Figure 15a, the 2f signal is maximal when the QCL wavelength matches the maximum of the NO absorption line at 1906.15 cm^−1^, which corresponds to a laser drive current of 531 mA. The previously determined experimental parameters (QCL drive current, wavelength modulation depth and frequency) are kept and the time constant of the lock-in is fixed at 1 s to perform a gas concentration calibration measurement. A gas mixture (NO in N_2_) is injected through with a total flow of 20 mL/min and different dilution ratios to make concentrations steps. The measurement lasts around 2 h, with a stable signal of the acoustic transducer (Figure 15b). 

**Figure 16:**
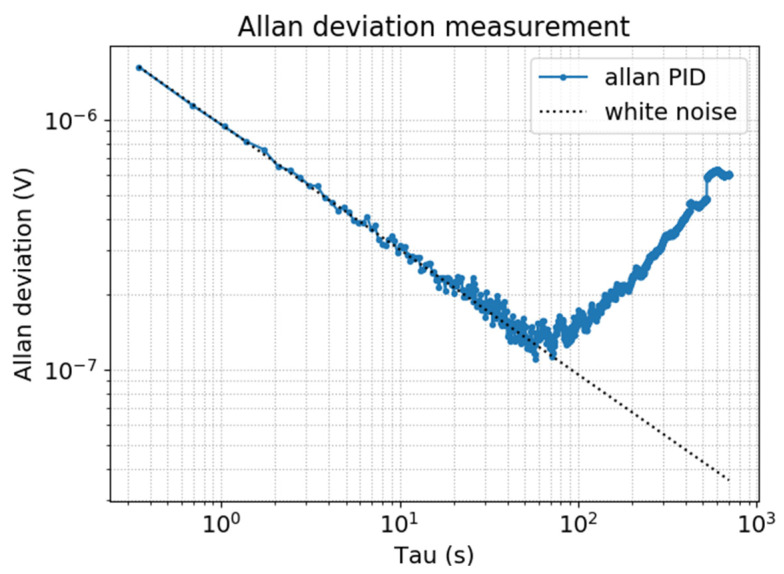
Allan deviation of the PA sensor in presence of a 5 ppm of NO in N_2_ (blue curve) and theoretical white noise curve in $1/\sqrt{t}$ (dotted black).

The step-by-step concentration measurement gives a linear sensor response and a sensitivity of 4.7 μV/ppm with a background noise of 11.7 µV. The noise measurement leads to a limit of detection (LOD) of $410\;\mathrm{ppb}/\sqrt{\mathrm{Hz}}$ and a normalized noise equivalent absorption (NNEA) of $5.1\times 10^{-7}\;W\cdot {\mathrm{cm}}^{-1}\cdot {\mathrm{Hz}}^{-1/2}$. The Allan deviation measurement (Figure 16) confirms a stability up to 58 s, which corresponds to a noise equivalent bandwidth of 1.3 mHz and thus an ultimate LOD of 15 ppb.

## 5. Conclusions

An all-optical multipass photoacoustic trace gas sensor, based on a Fabry–Perot hinged cantilever transducer, allowing remote detection and offering high sensitivity, has been presented. 

As a first step, we proposed a complete analytical lumped elements model to comprehensively describe the sensor’s frequency behavior and performances. With respect to the complete and lengthy FEM resolution, the model is much faster and provides however correct predictions. In addition, it can be easily transposed to different types and geometries of PA transducers. 

Then, we presented the fabrication process and the experimental results obtained on our Fabry-Perot acoustic transducer, characterized in a microphone configuration. Optical readout of the hinged cantilever displacement was performed with a 1.55 µm DFB laser. The mechanical structure was micromachined by laser cutting from a 100 µm stainless steel foil and assembled on a metallic support to create the FP cavity. The FP transducer has an optical cavity length of 1.6 mm and a mechanical resonance frequency at 5.1 kHz, for an acoustic LOD of $\sim \;2\;\mu \mathrm{Pa}/\sqrt{\mathrm{Hz}}$, which is comparable to noise floors of the current state-of-the-art optical microphones.

Based on this transducer, a PA sensor prototype, assembled with an “off-the-shelf” PA cell, has been proposed. The PA cell consists in a cylindrical multipass cavity with reflective walls to enhance the PA signal generation. A complete characterization of the FP based PA sensor was carried out with a QCL on a weak NO absorption line at 1906.15 cm^−1^. The measurements showed a good stability and a LOD of $410\;\mathrm{ppb}/\sqrt{\mathrm{Hz}}$, with a NNEA of $5.1\;\times \;10^{-7}\;W.{\mathrm{cm}}^{-1}.{\mathrm{Hz}}^{-1/2}$. Finally, we observed a good agreement between the experimental and calculated responses of the PA sensor.

In the near future, further improvements are expected, based on new prototypes with optimized PA chamber geometries and functioning at lower mechanical resonance frequency.

## Appendix A. Hinged Cantilever Effective Mass and Stiffness

The effective mass, stiffness and area of the first bending mode of the hinged cantilever are given by:

(A1) $$\{\begin{array}{c} k_{\mathrm{eff},m}=E_{m}\left(\int_{0}^{l_{1}}I_{1}{\left[\frac{d^{2}g_{1}\left(x\right)}{dx^{2}}\right]}^{2}dx+\int_{l_{1}}^{l_{1}+l_{2}}I_{2}{\left[\frac{d^{2}g_{2}\left(x\right)}{dx^{2}}\right]}^{2}dx\right)\\ m_{\mathrm{eff},m}=t_{m}\;\rho_{m}\left(\int_{0}^{l_{1}}w_{1}{\left[g_{1}\left(x\right)\right]}^{2}dx+\int_{l_{1}}^{l_{1}+l_{2}}w_{2}{\left[g_{2}\left(x\right)\right]}^{2}dx\right)\\ S_{\mathrm{eff},m}=t_{m}\;\left(w_{1}\int_{0}^{l_{1}}g_{1}\left(x\right)dx+w_{2}\int_{l_{1}}^{l_{1}+l_{2}}g_{2}\left(x\right)dx\right) \end{array},$$

where $g_{1}$ (resp. $g_{2}$) is the distribution function corresponding to the cantilever segment (resp. the hinge segment) and $w_{i}$, $l_{i}$, $I_{i}=\frac{w_{i}t^{3}}{12}$ are the width, length and moment of area of the considered segment:

(A2) $$g_{i}\left(x\right)=a_{i}+b_{i}x+c_{i}x^{3}\;\mathrm{with}\;\{\begin{array}{c} a_{1}=1\\ b_{1}=-\frac{3}{2}\;\frac{w_{2}l_{1}^{2}+w_{1}l_{2}\left(2l_{1}+l_{2}\right)}{A}\;\\ c_{1}=\frac{1}{2}\frac{w_{2}}{A} \end{array}\mathrm{and}\;\{\begin{array}{c} a_{2}=\frac{w_{1}{\left(l_{1}+l_{2}\right)}^{3}}{A}\\ b_{2}=-\frac{3}{2}\frac{w_{1}{\left(l_{1}+l_{2}\right)}^{2}}{A}\;\\ c_{2}=\frac{1}{2}\frac{w_{1}}{A} \end{array},$$

where $A=w_{2}l_{1}^{3}+w_{1}l_{2}\left(3l_{1}^{2}+3l_{1}l_{2}+l_{2}^{2}\right)$. The resonance frequency of the first bending mode is given by:

(A3) $$\omega_{m,\mathrm{hc}}=\sqrt{\frac{k_{\mathrm{eff},m}}{m_{\mathrm{eff},m}}}=\sqrt{\frac{w_{2}\left[l_{2}\left(3l_{1}^{2}+3l_{1}l_{2}+l_{2}^{2}\right)w_{1}+l_{1}^{3}w_{2}\right]}{B}}t\sqrt{\frac{E}{\rho_{m}}},$$

with $B=35l_{1}l_{2}^{2}\left(12l_{1}^{4}+30l_{1}^{3}l_{2}+33l_{1}^{2}l_{2}^{2}+18l_{1}l_{2}^{3}+4l_{2}^{4}\right)w_{1}^{2}+l_{2}\left(231l_{1}^{6}+273l_{1}^{5}l_{2}+105l_{1}^{4}l_{2}^{2}+63l_{1}^{2}l_{2}^{4}+91l_{1}l_{2}^{5}+33l_{2}^{6}\right)w_{1}w_{2}+33l_{1}^{7}w_{2}^{2}$.

## Appendix B. Lumped Model for the Heat Source

The F function is a solution of the Helmholtz equation:

(A4) $$\Delta F+k^{2}F=0\;\;\;\;\mathrm{with}\;\;\;k^{2}=\left(-𝕛\right)\frac{\rho_{0}\omega }{\kappa /c_{P}}\;\;\;\;\;\mathrm{and}\;r\in \partial V\;:F=1,$$

where κ is the thermal conductivity of air. The sidewall thermal losses are given by the $R_{r}$ impedance calculated with F(r,ω) considering an infinitely long cylinder and the upper and lower thermal losses are given by the $R_{z}$ impedance calculated with F(z,ω) considering an infinitely flat cylinder:

(A5) $$\{\begin{array}{c} F\left(r,\omega \right)=\frac{J_{0}\left(kr\right)}{J_{0}\left(kR_{cell}\right)}\;\mathrm{with}\;r\in \left[0,R_{\mathrm{cell}}\right]\\ F\left(z,\omega \right)=\frac{\cos\left(kz\right)}{\cos\left(\frac{kh_{cell}}{2}\right)}\;\mathrm{with}\;z\in \left[-h_{\mathrm{cell}}/2,h_{\mathrm{cell}}/2\right] \end{array}\;\Rightarrow \;\{\begin{array}{c} R_{r}=\frac{\rho_{0}c_{p}P_{0}}{8\kappa \pi }\frac{1}{h}\;\\ R_{z}=\frac{\rho_{0}c_{p}P_{0}}{8\kappa \pi }\frac{2h}{3R^{2}} \end{array}\;,$$

where $J_{0}$ denotes the Bessel function of first kind. The total thermal losses $R_{th}$ are given by the relation:

(A6) $$\frac{1}{R_{th}}=\frac{1}{R_{r}}+\frac{1}{R_{z}},$$
